# Trusting Social Media as a Source of Health Information: Online Surveys Comparing the United States, Korea, and Hong Kong

**DOI:** 10.2196/jmir.4193

**Published:** 2016-03-14

**Authors:** Hayeon Song, Kikuko Omori, Jihyun Kim, Kelly E Tenzek, Jennifer Morey Hawkins, Wan-Ying Lin, Yong-Chan Kim, Joo-Young Jung

**Affiliations:** ^1^ Gachon University Seongnam, Gyeonggi Province Republic Of Korea; ^2^ St. Cloud State University St. Cloud, MN United States; ^3^ Kent State University Kent, OH United States; ^4^ University of Buffalo Buffalo, NY United States; ^5^ University of Wisconsin-Milwaukee Milwaukee, WI United States; ^6^ City University of Hong Kong Hong Kong China (Hong Kong); ^7^ Yonsei University Seoul Republic Of Korea; ^8^ International Christian University Tokyo Japan

**Keywords:** social media, medical informatics, trust, culture, consumer behavior, consumer health information, information sharing

## Abstract

**Background:**

The Internet has increasingly become a popular source of health information by connecting individuals with health content, experts, and support. More and more, individuals turn to social media and Internet sites to share health information and experiences. Although online health information seeking occurs worldwide, limited empirical studies exist examining cross-cultural differences in perceptions about user-generated, experience-based information compared to expertise-based information sources.

**Objective:**

To investigate if cultural variations exist in patterns of online health information seeking, specifically in perceptions of online health information sources. It was hypothesized that Koreans and Hongkongers, compared to Americans, would be more likely to trust and use experience-based knowledge shared in social Internet sites, such as social media and online support groups. Conversely, Americans, compared to Koreans and Hongkongers, would value expertise-based knowledge prepared and approved by doctors or professional health providers more.

**Methods:**

Survey questionnaires were developed in English first and then translated into Korean and Chinese. The back-translation method ensured the standardization of questions. Surveys were administered using a standardized recruitment strategy and data collection methods.

**Results:**

A total of 826 participants living in metropolitan areas from the United States (n=301), Korea (n=179), and Hong Kong (n=337) participated in the study. We found significant cultural differences in information processing preferences for online health information. A planned contrast test revealed that Koreans and Hongkongers showed more trust in experience-based health information sources (blogs: *t*_451.50_=11.21, *P*<.001; online support group: *t*_455.71_=9.30, *P*<.001; social networking sites [SNS]: *t*_466.75_=11.36, *P*<.001) and also reported using blogs (*t*_515.31_=6.67, *P*<.001) and SNS (*t*_529.22_=4.51, *P*<.001) more frequently than Americans. Americans showed a stronger preference for using expertise-based information sources (eg, WebMD and CDC) compared to Koreans and Hongkongers (*t*_360.02_=3.01, *P*=.003). Trust in expertise-based information sources was universal, demonstrating no cultural differences (Brown-Forsythe *F*_2,654_=1.82, *P*=.16). Culture also contributed significantly to differences in searching information on behalf of family members (*t*_480.38_=5.99, *P*<.001) as well as to the goals of information searching.

**Conclusions:**

This research found significant cultural differences in information processing preferences for online health information. Further discussion is included regarding effective communication strategies in providing quality health information.

## Introduction

The Internet has increasingly become a popular source of health information by connecting individuals with health content, experts, and support. According to the Pew Internet & American Life Project [1], the Internet now exists as a popular venue for a number of health-seeking behaviors. For example, 35% of adults in the United States turn to the Internet to find information related to health and medical issues. Doctors and health professionals contribute to online health information, but a growing amount of health information on the Internet originates from individual patients sharing experiences. New digital platforms, such as social media and online support groups, allow a growing number of people to find peers who experience similar medical conditions or concerns, or to follow others’ experiences of health. For example, 26% of Internet users read or watched someone else’s experience about health or medical issues and 16% of Internet users deliberately tried to find others who experienced the same health concerns [2]. The Internet may serve a crucial role for individuals managing chronic illness, such as high blood pressure and diabetes, because one in four Internet users living with a chronic disease searched the Internet to find others with similar health concerns [2].

Research identifies two types of health information: expertise-based information produced by medical professionals and experience-based information based on laypersons’ subjective first-hand experiences of health and illness [3]. With the help of Web 2.0, user-generated, experience-based information emerges from “enormous knowledge assets that reside in collectives and communities” [4]. Further, experience-based information empowered by the Internet’s ability “to aggregate individuals’ experiences or opinion, pool their information, and identify the expertise of ‘nonexperts’ based on specific or situated knowledge” [5] currently challenges traditionally credentialed expertise. Some studies empirically investigate how Internet users differentially trust expertise- versus experience-based information sources. Eastin [6] found high-expertise source information tends to be perceived as more credible than low-expertise sources, whereas Hu and Sundar [7] did not find significant differences in the perceived credibility of messages prepared by doctors compared to laypersons.

Limited studies exist explaining such inconsistent findings—a gap remains in research examining possible factors affecting trust in expertise- versus experience-based health information available online. Further, it remains unknown whether clear cultural differences exist in perceptions about experience-based health information compared to expertise-based information sources. Because online health information seeking occurs worldwide [8] and health care is becoming more of a global issue [9], understanding cultural differences in online health information-seeking behavior becomes important. Are certain types of health information sought, trusted, and used more frequently in certain cultures compared to others? Do any cultural differences exist in terms of the effectiveness in communication strategies in providing health information online? Proper answers to these questions would be critical for professionals and community health workers serving multiethnic, multicultural global communities. Accordingly, this study compares data collected from the United States, South Korea, and Hong Kong to investigate if cultural variations exist in patterns of online health information seeking, specifically in perceptions of online health information sources.

### Cultural Emphasis on Experience-Based and Expertise-Based Information

Although various factors may affect differences across the United States, Korea, and Hong Kong, cultural theories provide a useful framework for understanding differences in the perception and seeking behaviors of online health information. According to Nisbett [10,11], culture influences information processing strategies and one’s general thoughts and beliefs. Nisbett and associates claim Easterners tend to possess a holistic approach, whereas Westerners predominately hold analytical and logical approaches [10-14]. A holistic approach involves an orientation to the context or field as a whole and a preference for explaining and predicting events based on such relationships [10]. Holism resists decontextualization, the separation of form from content, and the reliance on concrete instances and experiences. On the other hand, an analytic approach focuses on the categories to which an object belongs and relies on rules using formal logic. That is, such an approach includes a tendency to analyze the whole to determine key elements using logical understanding.

Several empirical studies have demonstrated Americans and Europeans are more likely to use logical, analytic, and rule-based reasoning, whereas East Asians are more likely to use intuitive, experience-based, and holistic reasoning [14,15]. Norenzayan and colleagues [14] compared the reasoning styles of European Americans and East Asians when participants were provided with a series of tasks (categorization, conceptual structure, and deductive reasoning) activating cognitive conflict. Findings suggest European Americans are more willing to set aside intuition and utilize rule-based reasoning than East Asians. The rationale is that individuals with an analytic approach possess a strong tendency to use abstract rules rather than experience for tasks of categorization and deductive reasoning. Moreover, Buchtel and Norenzayan [15] found Koreans (ie, those from a holistic culture) ranked intuition as more important than logic for success both at work and in relationship building. Further, East Asians rated an employee who made business decisions based on intuition higher than an employee who made decisions based on rule-based logic. Large-scale differences in cognitive preferences may influence various aspects of everyday life, including persuasion [16], trust building [17], and buying decisions [17].

Although not directly studied within a health-related context, extant research has demonstrated how cultural orientation influences people’s preferences for online information and trust building on the Internet across cultures. Access to experience-based information through word-of-mouth has been deemed an influential factor affecting consumer behaviors. Many marketing scholars have demonstrated the important role of customer reviews (ie, experience-based information) in e-commerce among various cultures [16,18,19]. For example, Utz et al [19] demonstrated that consumer reviews of stores had stronger effects on consumer behaviors than the overall reputation of the stores in the Netherlands. Lim et al [16] found the use of customer endorsement was more effective in building consumer trust in online shopping stores than the use of portal affiliation with stores in Hong Kong. Further, a study conducted in Taiwan specifically demonstrated that customer recommendations were more effective than expert recommendations on online product choices [18]. Going beyond a study in a single country, Sia and colleagues [17] conducted a comparative study about effects of customer endorsement and portal affiliation between two countries: Australia and Hong Kong. They found the impact of peer-customer endorsement on trust levels was stronger for individuals in Hong Kong than those in Australia. On the other hand, the effect of portal affiliation was more effective in Australia than Hong Kong.

Taken together, the previously mentioned studies imply that experience-based information shared online is becoming important across cultures and that Easterners rely more on it compared to Westerners. Particularly, findings imply that Easterners tend to show stronger trust toward those within their network (a whole), which is relevant to one of the core aspects of holism [12,14]. However, there is little evidence showing that this is also the case for health information seeking. One example is a study that indicates that people in the United States tend to seek information in online medical journals, whereas Japanese people prefer to find online health information in support groups [20].

Based on the previous empirical studies and the theoretical arguments that examine cultural differences in trust of online information, the goal of this study is to investigate cultural differences in trust of online health information. Specifically, we predict that Koreans and Hongkongers, compared to Americans, would be more likely to trust and use experience-based information shared in social Internet sites such as social media and online support groups. Conversely, Americans would be more likely to value health information prepared or approved by doctors or professional health providers (expertise-based information) than Koreans and Hongkongers.

Therefore, hypothesis 1a is Koreans and Hongkongers, compared to Americans, will report higher levels of trust in experience-based online health information sources (eg, social networking sites [SNS], blogs, online support groups). In contrast, hypothesis 1b is Americans, compared to Koreans and Hongkongers, will report higher levels of trust in expertise-based online health information sources (eg, WebMD).

Hypothesis 2a is Koreans and Hongkongers, compared to Americans, will use experienced-based sites more frequently. In contrast, hypothesis 2b is Americans, compared to Koreans and Hongkongers, will use expertise-based sites more frequently.

### Goals of Online Health Information–Seeking Behavior

In addition, we investigated cultural differences in the goals of online health information-seeking behavior to better understand preferences for experience-based and expertise-based information. Studies have suggested that several goals of online health information-seeking behavior differ before and after seeing a physician. Before meeting their doctor, patients go online mainly to (1) assess the need for consultation, (2) decide which physician to see, or (3) prepare for consultation [21,22]. After meeting the doctor, some patients might question the information provided to them by their doctor and decide to go online to get more information [23,24]. Patients may also turn to the Internet to better understand their diagnosis and treatment [21,22] and/or make sure they fully understand their health issues and have enough information [25]. Similarly, Caiata-Zufferey et al [26] categorized the goals related to online health information: (1) health maintenance, (2) preparing for consultation, (3) complementing consultation, and (4) validating/challenging consultation. Given that a significant number of people go online with various goals for health information seeking, it is important for educators, health care professionals, and website developers to further understand if cultural differences exist in types of goals for seeking health information. Thus, the following research question is raised: do cultural differences exist in goals of online health information-seeking behavior?

### Searching for Online Health Information on Behalf of Family Members

Individuals seek, find, and share health information online not only for themselves, but also for others, such as friends and family. Surprisingly, approximately half of all online health searches are performed on behalf of someone else [1]. For example, 53% of online health information seekers living with chronic diseases reported their last online health information search was related to the medical situation of someone else.

Although not much is known about the social aspects of online health information-seeking behaviors among Asians, some evidence suggests Asians may seek online health information on behalf of family members more frequently compared to Americans. Studies have found Asian and Latin American adolescents possess greater responsibilities in assisting, respecting, and supporting their families than their European counterparts [27]. Asian cultures often prioritize family along with values of obedience, duty, and in-group harmony [28,29]. We predict that Koreans and Hongkongers, compared to Americans, would possess a higher proclivity to search online health information on behalf of their family members. Therefore, our third hypothesis is cultural differences exist in searching for information for family members, such that Koreans and Hongkongers, compared to Americans, are more likely to search online health information on behalf of their family members.

## Methods

### Sample

The survey questionnaire for this study was developed in English first and then translated into Korean and Chinese. The back-translation method ensured the standardization of questions. In 2012, surveys were distributed to college students living in metropolitan areas of three different countries: the United States (Milwaukee, WI), South Korea (Seoul), and Hong Kong. Participants were solicited from large lectures at each university (University of Wisconsin-Milwaukee, Yonsei University, and City University of Hong Kong) using a standardized recruitment procedure and data collection method. Participation was voluntary. Required IRB documents were prepared and approved. An informed consent form was provided at the beginning of the survey.

### Measures

#### Health Information Seeking: Frequency and Trust

The frequency of using particular health information sources—blogs, support groups, SNS (eg, Facebook, Twitter), and professional health information websites (eg, WebMD, Centers for Disease Control and Prevention [CDC])—was measured with a range from 1 (never) to 7 (every day). Health information sources were modified for each country, allowing the list to reflect the most popular and representative sources, and subsequently verified by media statistics and media researchers living in each country. The level of trust in each health information source was also based on a 7-point Likert scale ranging from 1 (not at all) to 7 (completely).

#### Goals of Online Health Information Seeking

Four major goals of seeking online health information were developed and assessed based on a study by Caiata-Zufferey et al [26]: (1) health maintenance, such as “to maintain a healthy lifestyle” (α=.84); (2) preparation, such as “to determine whether I need to see a doctor” (α=.90); (3) complementing consultation, such as “after seeing my doctor to obtain more information” (α=.89); and (4) validating/challenging consultation, such as “to find different options for treatment” (α=.92). Responses were obtained on a 7-point Likert scale (1=strongly agree, 7=strongly disagree) with five items measuring each variable.

#### Seeking Health Information on Behalf of Family Members

The extent to which participants sought information on behalf of family members was measured by the level of agreement with the following statement: “Searching information for sick family members is an important family responsibility.” Responses were obtained using a 7-point Likert-type scale (1=strongly agree, 7=strongly disagree).

### Statistical Analysis

To test the hypotheses and research question, a series of 1-way ANOVAs were conducted followed by a planned contrast test. Before ANOVA testing, Levene’s test was conducted to check whether or not equal variance could be assumed. When the group variances were statistically equal, ANOVA *F* test was conducted. When equal variance could not be assumed, the Brown-Forsythe test was conducted instead of the ANOVA *F* test to reduce type I error. As a next step, a planned contrast test was conducted to systematically compare cultural differences. For the planned contrast test, the first level was to compare between analytic (ie, United States) and holistic (ie, Korea and Hong Kong) cultures, and then the second level was tested for a subsequent comparison between Korea and Hong Kong. The level of significance was set at .05.

## Results

A total of 826 native residents (301 in the United States, 179 in Korea, and 337 in Hong Kong) were included in the analysis (see Table 1). Among the 826 participants, 484 were male and 316 were female. Among US participants, 168 were male and 130 were female, whereas Korean participants consisted of 69 males and 104 females. The Hong Kong sample consisted of 245 males and 82 females. The overall mean participants’ age was 21.11 (SD 3.62) years. The participants from Hong Kong were slightly younger (mean 20.24, SD 2.88 years) than US students (mean 21.56, SD 4.66 years) and Korean students (mean 22.05, SD 2.36 years). Among US participants, a majority (83.2%, 248/298) were white followed by African American (6.4%, 19/298), Asian (3.7%, 11/298), and Hispanic/Latino (2.3%, 7/298). For the Hong Kong and Korean samples, close to 100% identified as Asian.

**Table 1:** Descriptive statistics of the participants.

Characteristics		United States (n=301)	South Korea (n=179)	Hong Kong (n=337)	Total (N=826)
**Gender, n (%)**					
	Male	168 (56.4)	69 (39.9)	245 (74.9)	482 (60.4)
	Female	130 (43.6)	104 (60.1)	82 (24.9)	316 (39.6)
Age (years), mean (SD)		21.56 (4.66)	22.05 (2.36)	20.24 (2.88)	21.11 (3.63)
**Internet access, n (%)**					
	Has Internet access	294 (98.0)	176 (100))	331 (98.2)	801 (98.5)
	Internet access through smartphone	177 (58.8)	176 (99.4)	321 (95.3)	674 (82.8)
**Internet use**					
	Daily Internet use, n (%)	296 (98.7)	175 (98.9)	330 (97.9)	801 (98.4)
	Hours using Internet/day, mean (SD)	4.44 (0.16)	2.92 (0.31)	4.38 (0.16)	4.23 (2.82)
**Health information**					
	Ever used Internet for health information, n (%)	270 (90.6)	167 (93.3)	261 (80.6)	698 (87.1)
	Frequency of online health information seeking, mean (SD)^a^	3.56 (1.11)	3.69 (1.13)	3.20 (0.89)	3.45 (1.06)

^a^ Frequency of online seeking measured with 7-point scale (1=never, 2=once a year, 3=couple of times a year, 4=once a month, 5=once a week, 6=2-3 times a week, and 7=every day).

A majority of individuals from each country had Internet access at home or at their primary place of residence, such as a dorm (United States: 98.0%, 294/300; Korea: 100%, 176/176; Hong Kong: 98.2%, 331/337). A majority of participants from Korea (99.4%, 176/177) and Hong Kong (95.3%, 321/336) reported they had mobile phones with an Internet connection, whereas only 58.8% (177/301) of the US sample reported having mobile phones with an Internet connection. Regardless of cultural background, most participants used the Internet daily (United States: 98.7%, 296/300; Korea: 98.9%, 175/177; Hong Kong: 97.9%, 330/337). In terms of the hours spent on the Internet each day, American university students used the Internet most often (mean 4.44, SD 0.16 hours) followed by students from Hong Kong (mean 4.38, SD 0.16 hours) and Korea (mean 2.92, SD 0.31 hours). Most participants reported using the Internet for health information and the frequency of online heath information seeking was from a couple of times a year to once a month.

The first hypothesis tested whether cultural differences exist in trust associated with the types of online health information sources, in particular experience-based online health information and expertise-based sites (see Tables 2-4). Regarding the first hypothesis, significant cultural differences were observed in the level of trust in all the experience-based sources, including blogs (Brown-Forsythe *F*_2,652_=74.91, *P*<.001), support groups (Brown-Forsythe *F*_2,627_=210.48, *P*<.001), and SNS (Brown-Forsythe *F*_2,621_=101.21, *P*<.001). Next, a planned contrast test was conducted to systematically compare the three countries. In the first level of the planned contrast analysis, all three experience-based sources showed significant cultural differences between analytic and holistic cultures such that individuals in the holistic culture compared to individuals in the analytic culture held higher levels of trust in SNS (*t*_466.75_=11.36, *P*<.001), blog (*t*_451.50_=11.21, *P*<.001), and online support groups (*t*_455.71_=9.30, *P*<.001). The additional planned contrast test (level 2) indicated Hongkongers, compared to Koreans, possessed significantly more trust in SNS, online support groups, and professional health sites. No significant cultural differences were detected in the level of trust in expertise-based sources, including online professional health sites (Brown-Forsythe *F*_2,654_=1.82, *P*=.16). Thus, hypothesis 1a was supported, but hypothesis 1b was not.

**Table 2:** Cultural differences of the trust level in each source of online health information.

Internet sites	Country, mean (SD)			Brown-Forsythe^a^		Planned contrast			
	United States	Korea	Hong Kong	*F* (*df*)	*P*	Level 1: US vs KOR/HK		Level 2: KOR vs HK	
						*t* (*df*)	*P*	*t* (*df*)	*P*
SNS	2.30 (1.43)	3.16 (1.18)	3.79 (1.04)	101.21 (2, 621)	001	11.36 (467)	.001	5.76 (316)	.001
Blog	2.86 (1.39)	4.04 (1.04)	3.90 (1.04)	74.91 (2, 652)	.001	11.21 (452)	.001	1.29 (352)	.20
Online support groups	3.34 (1.49)	3.34 (1.18)	5.32 (1.06)	210.48 (2, 627)	.001	9.30 (456)	.001	17.78 (323)	.001
Online professional heath sites^b^	5.54 (1.25)	5.39 (1.10)	5.61 (1.13)	1.82 (2, 654)	.16	.42 (511)	.68	1.98 (355)	.05

^a^ For 1-way ANOVA test, we used Brown-Forsythe because equal variances could not be assumed. Thus, *F* value in ANOVA indicates asymptotically *F* distributed.

^b^ Expertise-based source.

**Table 3:** Cultural differences in the frequency of using each source of online health information.

Internet sites	Country, mean (SD)			Brown-Forsythe^a^		Planned contrast			
	United States	Korea	Hong Kong	*F* (*df*)	*P*	Level 1: US vs KOR/HK		Level 2: KOR vs HK	
						*t* (*df*)	*P*	*t* (*df*)	*P*
SNS	2.16 (1.51)	2.27 (1.21)	3.07 (1.52)	32.25 (2, 697)	.001	4.51 (529)	.001	6.06 (405)	.001
Blog	2.50 (1.47)	3.61 (1.37)	2.85 (1.25)	34.61 (2, 622)	.001	6.67 (515)	.001	5.82 (326)	.001
Online support groups	2.26 (1.54)	2.15 (1.16)	2.74 (1.46)	12.40 (2, 698)	.001	1.64 (497)	.10	4.70 (409)	.001
Online professional heath sites^b^	4.68 (1.62)	2.66 (1.37)	3.08 (1.45)	122.57 (2, 664)	.001	15.02 (508)	.001	360.02 (360)	.003

^a^ For 1-way ANOVA test, we used Brown-Forsythe because equal variances could not be assumed. Thus, *F* value in ANOVA indicates asymptotically *F* distributed.

^b^ Expertise-based source.

Hypothesis 2a-b investigated a usage pattern of each online health information source, in particular experience-based sites and expertise-based sites (see Tables 3 and 4). Significant cultural differences existed in the frequency of utilizing experience-based information sources (eg, blogs, support groups, SNS) as well as expertise-based sources (ie, online professional health sites). Specifically, results of the 1-way ANOVA test for experience-based knowledge information sources were significant: blogs (Brown-Forsythe *F*_2,622_=34.61, *P*<.001), support groups (Brown-Forsythe *F*_2,698_=12.40, *P*<.001), and SNS (Brown-Forsythe *F*_2,697_=32.25, *P*<.001). The planned contrast test revealed individuals in a holistic culture used blogs (*t*_515.31_=6.67, *P*<.001) and SNS (*t*_529.22_=4.51, *P*<.001) significantly more than individuals in an analytic culture. However, no significant cultural differences were found in terms of online support group use (*t*_455.71_=1.64, *P*=.10). A subsequent planned contrast test (level 2) found significant differences between Koreans and Hongkongers in the use of all four types of information sources. Overall, findings indicate partial support for hypothesis 2a.

**Table 4:** Cultural differences in the frequency of using each source of online health information.

Source and value label^a^		United States		Korea		Hong Kong	
		n (%)	Cumulative %	n (%)	Cumulative %	n (%)	Cumulative %
**SNS**							
	1	132 (48.5)	48.5	55 (33.1)	33.1	51 (18.6)	18.6
	2	64 (23.5)	72.1	53 (31.9)	65.1	59 (21.5)	40.1
	3	17 (6.3)	78.3	25 (15.1)	80.1	52 (19.0)	59.1
	4	31 (11.4)	89.7	24 (14.5)	94.6	64 (23.4)	82.5
	5	19 (7.0)	96.7	9 (5.4)	100.0	33 (12.0)	94.5
	6	5 (1.8)	98.5			10 (3.6)	98.2
	7	4 (1.5)	100.0			5 (1.8)	100.0
**Blog**							
	1	92 (17.8)	17.8	9 (2.4)	2.4	38 (2.6)	2.6
	2	67 (27.5)	45.4	37 (4.8)	7.2	85 (9.2)	11.7
	3	39 (22.7)	68.0	27 (18.6)	25.7	60 (13.2)	24.9
	4	45 (19.0)	87.0	40 (39.5)	65.3	64 (48.4)	73.3
	5	20 (9.7)	96.7	45 (30.5)	95.8	21 (23.8)	97.1
	6	7 (2.2)	98.9	9 (4.2)	100.0	5 (2.9)	100.0
	7	2 (1.1)	100.0				
**Support group**							
	1	123 (45.7)	45.7	59 (35.3)	35.3	64 (23.5)	23.5
	2	57 (21.2)	66.9	59 (35.3)	70.7	76 (27.9)	51.5
	3	30 (11.2)	78.1	21 (12.6)	83.2	46 (16.9)	68.4
	4	32 (11.9)	90.0	21 (12.6)	95.8	52 (19.1)	87.5
	5	12 (4.5)	94.4	7 (4.2)	100.0	24 (8.8)	96.3
	6	12 (4.5)	98.9			6 (2.2)	98.5
	7	3 (1.1)	100.0			4 (1.5)	100.0
**Professional** ^b^							
	1	11 (4.1)	4.1	41 (24.8)	24.8	41 (15.0)	15.0
	2	17 (6.3)	10.3	45 (27.3)	52.1	66 (24.1)	39.1
	3	35 (12.9)	23.2	29 (17.6)	69.7	62 (22.6)	61.7
	4	58 (21.4)	44.6	32 (19.4)	89.1	59 (21.5)	83.2
	5	53 (19.6)	64.2	15 (9.1)	98.2	33 (12.0)	95.3
	6	59 (21.8)	86.0	3 (1.8)	100.0	8 (2.9)	98.2
	7	38 (14.0)	100.0			5 (1.8)	100.0

^a^ For value label: 1=never, 2=rarely, 3=sometimes, 4=moderately, 5= fairly often, 6=often, and 7=always.

^b^ “Professional” indicates professional online health sites, such as WebMD and CDC. This is also an expertise-based source.

The result of the overall test for expertise-based information was significant (Brown-Forsythe *F*=233.57, *P*<.001). The planned contrast test result (level 1) suggested significant cultural differences between analytic and holistic cultures (*t*_508.47_=15.02, *P*<.001) indicating Americans searched expertise-based health information (mean 4.68, SD 1.20) significantly more often than participants from Hong Kong (mean 3.08, SD 1.45) and Korea (mean 2.66, SD 1.37). The following level of planned contrast test (level 2) showed the difference between Hongkongers and Koreans was also significant (*t*_360.02_=3.01, *P*=.003). Overall, hypothesis 2b was supported. Additional analyses were conducted to determine whether or not offline health information–seeking behavior is similar to that of online health information–seeking behavior. The frequency of using a health care provider as a source of health information showed significant results for both overall 1-way ANOVA test (Brown-Forsythe *F*=57.23, *P*<.001) and the planned comparison test (*t*_533.28_=3.71, *P*<.001) indicating Americans consulted health care providers to a significantly greater extent than Koreans and Hongkongers.

Regarding the third hypothesis, the result of the 1-way ANOVA test for online health information–seeking behavior on behalf of family was significant (Brown-Forsythe *F*=27.74, *P*<.001). The planned contrast test (level 1) revealed significant differences between the United States (mean 4.25, SD 1.57) and Hong Kong (mean 4.61, SD 1.25) and Korea (mean 5.25, SD 1.25; *t*_480.38_=5.99, *P*<.001). Thus, participants from holistic cultures were more likely to perceive searching for health information on behalf of a family member was an important family responsibility compared to participants from an analytic culture. At the same time, a significant difference existed between Hong Kong and Korea (*t*_338.08_=5.16, *P*<.001). Overall, hypothesis 3 was supported.

Research question 1 asked whether cultural differences existed in the goals of online health information–seeking behaviors. Findings indicated significant cultural differences in the goals of health maintenance (Brown-Forsythe *F*=8.43, *P*<.001) with significant differences observed between the United States (mean 3.50, SD 1.33) and Asian countries (Hong Kong: mean 3.22, SD 0.99; Korea: mean 3.06, SD 1.10; *t*_473.54_=3.75, *P*<.001). Regarding preparing for consultation, the result of the 1-way ANOVA test was significant (*F*_2,691_=35.56, *P*<.001, η^2^=0.09). Further, the goal of preparing for consultation was higher among Americans (mean 3.58, SD 0.08) than among Asians (Hong Kong: mean 2.83, SD 0.08; Korea: mean 3.85, SD 0.11; *t*_691_=2.30, *P*=.02). The goal of complementing a consultation also showed significant differences among the three countries (*F*_2,698_=7.43, *P*=.001, η^2^=0.02). The planned comparison test revealed Asians (Hong Kong: mean 3.49, SD 0.08; Korea: mean 3.40, SD 0.10), compared to Americans (mean 3.09, SD 0.08), tended to engage in health information seeking to a greater extent to complement a health consultation, demonstrating an opposite pattern with the aforementioned two goals. In terms of the goal of health information seeking to challenge a consultation, no significant differences were found among the three countries (*F*_2,689_=0.87, *P*=.42).

## Discussion

More individuals are turning to social media to share health information and experiences these days. While online, individuals can easily and efficiently find other individuals who have similar health concerns or experiences. This study sheds light on the experience-based health information commonly shared on social sites, such as blogs, SNS, and online health support groups. Specifically, we examined individuals’ trust in experience-based health information presented on social sites compared to their trust in expertise-based health information found on professional sites. As expected, peer-to-peer exchange of experience-based health information online was popular: 51.5% of Americans, 76.9% of Koreans, and 81.4% of Hongkongers reported using SNS for health information, whereas 66.2% of Americans, 94.6% of Koreans, and 86.1% of Hongkongers reported using blogs for health information.

Although social Internet sites function as important online health information sources across cultures, we found significant cultural differences in preferences for types of information found and shared on the Internet. Based on theoretical underpinnings of Nisbett’s cultural theory, we hypothesized Koreans and Hongkongers, compared to Americans, would be more likely to trust and use social Internet sites, such as blogs, social support groups, and SNS. The hypothesis was supported. In addition, as we expected, the study’s findings indicate that expertise-based health information sites are used more frequently by Americans than Koreans and Hongkongers (no country-level differences were detected in terms of trust in expertise-based health information sites). The findings resonate with previous studies demonstrating that Asian cultures, which are predominantly holistic, are more likely to value experience-based information, whereas Western cultures are more likely to value logical expertise- and rule-based information. In addition, we also observed cultural differences in searching for information on behalf of family members. As expected, participants from holistic cultures (Korea and Hong Kong) sought information for family members more than participants from an analytic culture (United States) did.

Regarding information-seeking behaviors in the offline context, Americans generally trusted and used offline sources, including both experience- and expertise-based sources. Further, pairwise comparison revealed that Hongkongers trusted information from laypersons, such as family and friends, more strongly than Americans did, whereas Americans trusted information from health professionals more significantly than Hongkongers. However, no differences were found between Americans and Koreans. When engaging in actual information seeking, Hongkongers consulted both family/friends and health professionals significantly less than did both Americans and Koreans. This finding may suggest the Internet’s strengths in tailoring to meet individual needs and cognitive preferences. The Internet is a proficient medium for audience segmentation in that it efficiently finds people who hold similar interests or concerns [30]. When inquiring about health information offline, social networks prove less useful in locating individuals who share the same health concerns or problems. Consulting a wider online social network may produce higher chances of finding someone who shares rare or specific health concerns or questions. With this structural feature, the Internet may better meet needs based on cultural differences.

This study offers several practical implications for the dissemination of health information online. First, our study confirms that experience-based health information is widely used across countries; therefore, professional health information providers should consider actively taking advantage of social media and similar applications when sharing information with patients (eg, providing examples of patients’ experiences). Leveraging social media or similar tools as the source of experience-based information can “increase access to, enliven users’ experiences with, and enrich the quality of the information available” [4]. Specifically, social media can help disseminate expertise-based health information by enhancing access, relevance, and credibility [4]. Habitual users of social media can access expertise-based information posted to social media sites with greater ease; moreover, the information accessed may be perceived as more relevant due to viewing the first-hand experiences of others in their networks. In addition, experience-based information shared on social media sites can strengthen the credibility of professional health information, which may be particularly true for East Asians.

Second, online health interventions targeting individuals from different cultural orientations should not discount differential cognitive preferences in locating effective communication strategies for providing health information online. Experience-based information can be strategically and differentially incorporated into expertise-based health information to target audiences from diverse cultures. For example, when designing health-related social media forums with expert moderators, stronger focus on rich, experience-based information should be included for Korean and Hong Kong audiences, whereas the expert role should be more pronounced for American audiences. Because perceived credibility is related to intentions to revisit websites [31], tailoring message sources and media environments may be helpful to ensure the success of providing health information across cultures.

Third, the current findings about cultural differences may also inform interactions in the offline context. Previous research with Korean participants illustrates that even though participants indicated a preference for physician interactions, only 10.9% of respondents with a health concern actually went to the physician first, whereas 48.6% indicated they consulted the Internet [32]. Thus, when communicating with Koreans and Hongkongers in medical settings or through health campaign messages, health care professionals and practitioners should maintain an awareness of a strong preference for experience-based knowledge. For example, in discussing treatment options, health care practitioners could ask patients about any experience-based knowledge found online. At the same time, a systematic review of online settings where experience-based knowledge is shared may need to be planned and designed by health professionals to validate the information. Although sharing experiences with similar health problems can be a great information resource for users, such information can be inaccurate or applied to the wrong situations. Professionals using experience-based knowledge strategically would likely produce a synergistic effect.

Lastly, we found, across cultures, Internet users possess different motivations for seeking health information online based on differing goals for the outcome of the search. Koreans and Hongkongers seek online health information primarily to make critical health decisions, such as whether to follow doctor’s instructions, whereas the primary goal of health information seeking for Americans is health maintenance and preparation for the medical consultation. In other words, inaccuracy or the incorrect application of information may be more critical among Koreans and Hongkongers than Americans due to goal differences. Incorporating health professionals’ comments in health-related blogs, SNS, and support group sites may be imperative for East Asian populations. To address Americans’ concerns related to health maintenance and medical consultation, key messages related to preventive health can be beneficial in promoting quality of life and cutting medical costs for Americans [9].

### Limitations

Although the study offers several significant contributions, some limitations exist. First, although this study presents data gathered from three different countries, research should focus on extending this work to other countries. Even though both Korea and Hong Kong are considered to be holistic cultures, significant differences still exist. This finding suggests that even though a dichotomous approach to culture bears differences, the cultural separation in beliefs extends beyond two categories. Future studies should include individual-level comparisons in addition to a country-level investigation. Additionally, factors affecting national differences, such as the level of institutional trust [33] or other structural distinctions, need to be further investigated.

Second, the sample consisted of participants who were relatively young, with a mean age of 21 years (SD 3.63); participants in all three countries were university students. Given that young people remain less likely to encounter serious health problems, the patterns observed in online health information-seeking behaviors may not replicate in older age groups. Similarly, because a sample of university students represents a highly educated group, individuals with different education levels or technology efficacy may demonstrate different perceptions and behaviors. For example, previous research indicates that individuals who have lower education levels are not as likely to search for health information online [32]. Health literacy and digital divide concerns are also part of the broader social conversation when it comes to barriers to online health information-seeking behaviors [34,35]. Therefore, future studies should further test the proposed hypotheses of this study on other populations, such as individuals with serious health problems, less education, and those of an older age.

Lastly, in addition to a theory-based explanation for cultural differences between the East and West, other factors might influence individual’s perceptions of online health information credibility and trust across cultures. For example, given the higher degree of ethnic homogeneity of the population in Korea and Hong Kong compared to the United States, it is plausible that individuals in these countries are more likely to be exposed to online health information generated by “people like them.” *Homophily*, or the degree of perceived similarity that a receiver ascribes to a message source, has been cited as a factor influencing individuals’ perceptions of online health information [36-38]. Wang and colleagues [38] examined how individuals evaluate health information from experts on websites compared to peers in online discussion groups. Their results indicate that when evaluating the health information offered in a discussion group, individuals who perceived stronger homophily reported a more positive evaluation of the information, which consequently led to greater likelihood of acting on the advised information. Similarly, when evaluating information presented on a website, the degree of perceived homophily also directly influenced perceived credibility and positive evaluations of the health information provided [38]. What remains unknown is whether cultural variations exist in the degree homophily is experienced, given the fact that homophily can be motivated by demographic factors (eg, age, ethnicity) and experience/attitudinal factors (eg, sharing emotion, attitude, and experience) [39]. Whether Koreans and Hongkongers, compared to Americans, tend to feel stronger homophily warrants further investigation in the context of online health information seeking.

### Conclusions

In conclusion, this study contributes to the literature on online health information–seeking behaviors by demonstrating a tendency for Koreans and Hongkongers to trust and use experience-based knowledge to a greater extent than Americans. Additionally, Koreans and Hongkongers are more likely to search for health information on behalf of family members, resonating with a holistic worldview. Cultural differences also exist in the goals associated with online health information. Asians engage in health information–seeking behavior to make health care decisions, an extremely important finding to consider when evaluating the credibility and trust of health information online. To achieve health and facilitate positive, peer-to-peer communication of health information, clinicians and scholars should continue to be aware of online health information–seeking behaviors before and after medical consultation and provide patients with avenues to navigate online sources. Similarly, health messages should also focus on cultural orientation to provide quality health care.

## Abbreviations

SNS: social networking site
